# Indigenous bacteria as an alternative for promoting recycled paper and cardboard mill wastewater treatment

**DOI:** 10.1038/s41598-022-21362-6

**Published:** 2022-10-06

**Authors:** Maryam Gholami, Mohammad Taghi Ghaneian, Fahimeh Teimouri, Mohammad Hassan Ehrampoush, Abbasali Jafari Nadoushan, sara Jambarsang, Amir Hossein Mahvi

**Affiliations:** 1 Environmental Science and Technology Research Center, Department of Environmental Health Engineering, School of Public Health, Shahid Sadoughi University of Medical Sciences, Yazd, Iran; 2 Environmental Science and Technology Research Center, Department of Environmental Health- Radiation Health, School of Public Health, Shahid Sadoughi University of Medical Sciences, Yazd, Iran; 3 Department of Medical Parasitology and Mycology, School of Medicine, Shahid Sadoughi University of Medical Sciences, Yazd, Iran; 4 Center for Healthcare Data Modeling, Departments of Biostatistics and Epidemiology, School of Public Health, Shahid Sadoughi University of Medical Sciences, Yazd, Iran; 5 Center for Water Quality Research (CWQR), Institute for Environmental Research, Tehran University of Medical Sciences, Tehran, Iran; 6 Center for Solid Waste Research, Institute for Environmental Research, Tehran University of Medical Sciences, Tehran, Iran

**Keywords:** Biological techniques, Environmental sciences

## Abstract

The present study aimed to investigate indigenous bacteria possibility in recycled paper and cardboard mill (RPCM) wastewater treatment through the isolation and identification of full-scale RPCM indigenous bacteria. The molecular characterization of the isolated bacteria was performed by 16S rRNA gene sequencing*. Klebsiella pneumoniae* AT-1 (MZ599583), *Citrobacter freundii* AT-4 (OK178569), and *Bacillus subtilis* AT-5 (MZ323975) were dominant strains used for RPCM wastewater bioremediation experiments. Under optimal conditions, the maximum values of chemical oxygen demand (COD) and color biodegradation by *C. freundii* AT-4 were 79.54% and 43.81% after 10 days of incubation, respectively. In the case of *B. subtilis* strain AT-5 and *K. pneumoniae* AT-1, the maximum values of COD and color biodegradation were 70.08%, 45.96%, 71.26%, and 32.06%, respectively. The results from optimal conditions regarding efficiency were higher in comparison with the efficiency obtained from the oxidation ditch treatment unit in full-scale RPCM-WWTP. Therefore, the present study introduces the isolated indigenous bacteria strains as a promising candidate for improving the RPCM-WWTP efficiency using bioremediation.

## Introduction

The pulp and paper industries have been among the major water consumers. Therefore, high water usage produces large volumes of highly colored and toxic wastewater^1,2^. Based on raw materials, there are two types of the paper industry, including recycled old paper and virgin pulp paper^3^. The recycled paper mill consumes approximately 20 m^3^ of water per ton of recycled paper and produces 18 m^3^ of wastewater. The characteristics of wastewater vary depending on the production method, the raw materials, and applied chemicals in the process^4^. Compared to wood pulp and paper mills, recycled paper mills and those with a water recycling system have more microbial and organic pollution due to outdoor exposure of recycled papers and water reuse^5^. Treatment, along with the removal of major pollutants is highly necessary from environmental protection perspectives^4,6–8^.

Various physical, chemical, and biological treatment methods include adsorption^2,3,7–9^, microfiltration^8^, membrane filtration^10^, reverse osmosis^3,9^, coagulation and flocculation^2,3,7–9,11^, electrical coagulation^7,10^, electrolysis^9^, chemical precipitation^2,7^, ion exchange^7^, oxidation^8,9,11^, ozonation^3,8,9,11^, fenton^9^, photo fenton^9^, and electro fenton^10^. However, none of these methods have been developed to be industrially applicable due to high costs, high sludge production, or other drawbacks^2,11^. The biological treatment methods can also be extremely ineffective when the effluent contains significant amounts of color, resistant organic compounds, and suspended solids^1,2^.

Biotechnological strategies such as bioremediation have recently received attention. Bioremediation is explained as an approach of using microorganisms to decompose or immobilize contaminants. The detoxification process converts the harmful materials to harmless end products by mineralization, transformation, or alteration mechanisms^12,13^.

Various studies have been performed to identify microorganisms capable of degrading resistant organic compounds^14^. Some studies have reported that certain groups of microorganisms, including *Bacillus megaterium*, *Serratia liquefaciens, Bacillus* sp*.*, *Bacillus subtilis, Pseudomonas stutzeri*, *Ancylobacter aquaticus, Klebsiella, Methylobacterium* spp., *Pseudomonas,* and *Comamonas*, are effective in degrading resistant organic compounds, lignin, and chlorinated organic compounds from wastewater^1,15–17^.

Although significant efforts have been made to use prepared isolated microorganisms, less attention has been given to examining the application of recycled paper and cardboard mill (RPCM) native microorganisms. Accordingly, the present study mainly sought to investigate the potential of indigenous bacteria in the RPCM wastewater treatment industry in promoting biological wastewater treatment, which has been performed as batch-scale experiments.

## Materials and methods

### RPCM treatment plant description and sampling

**Figure 1:**
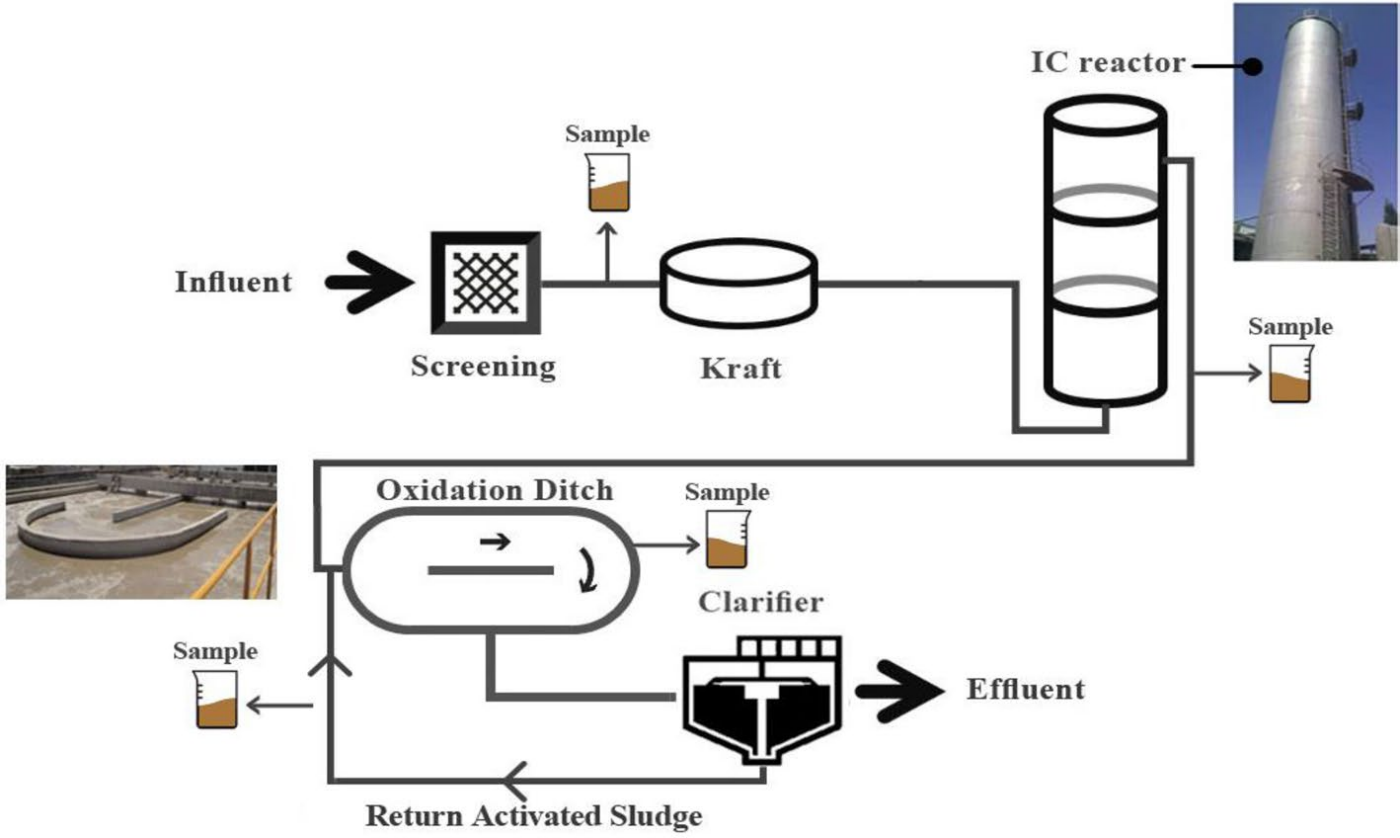
Schematic diagram of full-scale RPCM-WWTP. *Note*. RPCM: Recycled paper and cardboard mill; WWTP: Wastewater treatment plant.

RPCM industry is located in Yazd (Iran) with the aim of producing various types of packaging paper from recycled materials and has a production capacity of 270 ton/d. The RPCM wastewater treatment plant (RPCM-WWTP) was designed to treat 3000 m^3^/d of wastewater with an organic loading rate (OLR) of 12,000 kg COD/d. Water consumption and wastewater production equal 4.5–5 m^3^/ton.d and 3–3.5 m^3^/ton.d, respectively. The mean removal efficiency of chemical oxygen demand (COD) in the oxidation ditch unit of RPCM-WWTP equals 60.57 ± 16.73%. In addition, the latter biological treatment unit has not been effective in removing color. The arrangement of the RPCM-WWTP and sampling points is in Fig. 1.

As shown, to determine the characterization of communities of heterotrophic bacteria, grab sampling was performed from the main locations, including raw wastewater, internal circulation (IC) reactor, oxidation ditch, and return activated sludge. Then, the samples were immediately transferred to the laboratory into 500-mL sterile glass bottles.

### Isolation and bacterial identification

It was theorized that bacteria isolated from their natural habitats can survive in hard conditions by creating several catabolic enzyme systems. Thus, RPCM-WWTP bacteria were isolated and distinguished as an alternative for the improvement of WWT efficiency.

The collected samples were diluted by the serial dilution method (dilution series 10^–1^ to 10^–7^)^11^. Then, 0.1 mL of these solutions was cultured on plates containing a nutrient agar medium^16^. Next, the plates were incubated at 37 °C for 48 h^8^. The dominant bacterial colonies (dominant in this study was defined as the highest number of a colony on the plate) were selected and underwent purification. Meanwhile, the isolation process was performed by transferring a supposed colony section on a fresh nutrient agar plate using a sterile loop and culturing as the streak method to achieve pure colonies. Subsequently, the isolated colonies were labeled as RW1-RW4, IC1-IC3, AT1-AT5, and AS1-AS3. Then, the Gram-staining method was used to confirm the purity of the isolated dominant colonies, followed by determining the biochemical characterization of the isolated gram-negative dominant bacterial strains^18^.

In total, 15 bacteria strains were initially isolated from RPCM-WWTP. The isolated bacterial colonies were diverse in their morphologies. The molecular characterization of the isolated bacteria was performed by 16S rRNA gene sequencing. In addition, DNA was extracted using the boiling method. A NanoDrop spectrophotometer (Thermo scientific LR20-36) was used to evaluate the quantity and quality of the extracted DNA. Then, DNA templates were amplified by the polymerase chain reaction (PCR) using 16S rRNA universal primer set 27F (5′-AGAGTTTGATCCTGGCTCAG-3′) and 1492R (5′TACGGTTACCTTGTTACGACTT-3′)^8^. The 20 µL PCR mixture contained 10 µL of Master Mix, 7 µL of deionized water, 1 µL of each primer, and 2 µL of DNA template. The thermal cycling steps were 95 °C for 5 min (denaturation), followed by 30 cycles at 94 °C for 30 s, 55 °C for 35 s (annealing), 72 °C for 1 min (extension), and a final extension step at 72 °C for 5 min (Thermal cycler: Simpliamp, ABI, American). Electrophoresis (FANAVARANE SAHAND AZAR, Iran) and gel dock (compact, Iran) were employed to identify the quality of the DNA strand product. The PCR products were purified by the ExoSAP protocol, and the sequencing was performed using an Applied Biosystems 3500 (ABI) sequencer (Pishgam Biotech Co., Tehran, Iran). To determine the nearest homolog of the isolated bacterial strains, sequence data were analyzed in the BLAST tool (http://www.ncbi.nlm.nih.gov). According to the maximum identity score, the first few sequences were selected and aligned by jPHYDIT software (version 1.0). The retrieved sequences were submitted to Bankit, and the accession numbers were received for the identified isolates^19,20^. Finally, the phylogenetic tree was plotted using jPHYDIT software (version 1.0).

### Bioremediation experiments

#### Culture preparation

Overall, three bacterial strains out of 15 isolates, including *Klebsiella pneumoniae* (*K. pneumoniae*) AT-1 (MZ599583), *Citrobacter freundii* (*C. freundii*) AT-4 (OK178569), and *Bacillus subtilis* (*B. subtilis*) AT-5 (MZ323975), were used for bioremediation setups (based on their fast growth in the nutrient agar medium and COD and color removal potential).

#### Experimental setups

A homogenous suspension (as culture) containing 1.5 × 10^8^ CFU/mL (0.5 McFarland) of each bacterium was prepared and separately used for bioremediation. The experiments were performed in conical flasks (250 mL) containing 150 mL of the effluent and culture. Table 1 presents the experimental setup condition of bacterial strains.

**Table 1:** Experimental setup conditions.

Setup NO	Effluent	Culture (%, v/v)	Mineral salt	Glucose	Consideration
1	√	10	–	–	
1	√	–	–	–	Control
2	√	10	√	–	
2	√	–	√	–	Control
3	√	10	√	√	
3	√	–	√	√	Control

Based on data in Table 1, mineral salts (Na_2_HPO_4_, 2.4 g/L; K_2_HPO_4_, 2.0 g/L; NH_4_NO_3_, 0.1 g/L; MgSO_4_, 0.01 g/L; CaCl_2_, 0.01 g/L) and glucose (0.5%, w/v) were used as co-substrates. The solution pH was adjusted (pH = 7.2) and sterilized for 20 min at 121 °C^14^. Glucose was added after sterilization using a sterile syringe filter (20 µm). The inoculated flasks were incubated in a shaker incubator at 140 rpm and 30 °C for 10 days.

The influent of the oxidation ditch reactor (corresponding to COD = 1056 mg/L and color = 610 ADMI; The American Dye Manufacturer’s Institute) was investigated to perform bioremediation experiments. Colony forming unit (CFU/mL) counts method were performed to measure bacterial growth by the serial dilution method^14^. The samples were withdrawn on days 2, 4, 6, and 10 for the analysis of bacterial growth, periodic monitoring of operating parameters, and reduction in COD and color.

#### Chemicals and physicochemical analyses

Culture media and chemicals were of analytical grade and purchased from Merck Company. All parameters were analyzed according to Standard Methods for the Examination of Water and Wastewater Instruction^18^. The pH parameter was measured by a pH meter (L2012, Labtron Co, Iran). The turbidity meter was used to determine the turbidity (A-TUR-1.16, AndisheSazan Electricity Industry, Iran), and the ADMI method was utilized to measure the color (DR6000, HACH, USA). Further, COD was measured by the closed reflux method (Method: 5220-D). Furthermore, dissolved oxygen (DO), electrical conductivity (EC), and temperature were determined by a multi-meter (HQ40d, HACH, USA). Bioremediation experiments were conducted in triplicate, and the data are presented as the mean ± standard deviation.

## Results and discussion

### RPCM industrial wastewater characteristics

Six samples in three months were analyzed to investigate the qualitative characteristics of RPCM raw wastewater. Table 2 provides the main characteristics of raw wastewater. RPCM influent wastewater had high turbidity, total suspended solids (TSS), color, and COD, along with a dark brown color and pH ≈ 6.4 (Table 2). Previous studies have reported that pulp and paper wastewater had high levels of COD, lignin, and cellulose, while pH was in the neutral range^9,17,21,22^. The discharge of effluents containing these compounds has adverse effects on receiving aquatic environments^9,23^.

**Table 2:** Characteristics of RPCM raw wastewater.

Parameter	Sample						Average values
Sampling frequency	1	2	3	4	5	6	
pH	6.31	6.34	6.51	6.59	6.17	6.23	6.36 ± 0.16
EC (ms/cm)	10.07	9.29	8.4	8.66	9.43	8.59	9.07 ± 0.67
(°C) Temperature	26.6	26.9	27.1	26.5	27	26.7	26.8 ± 0.24
(NTU) Turbidity	4140	2940	4960	3550	2380	2320	3381.67 ± 1041
TSS (mg/L)	6100	3770	4740	1550	2460	2600	3536.67 ± 1676
(ADMI) Color	1300	1460	1580	1200	1400	1400	1390 ± 130
COD (mg/L)	9033	8950	8116	6485	6602	6567	7625.7 ± 1220

RPCM, recycled paper and cardboard mill.

### Identification of dominant bacterial strains

#### Isolation and phenotype identification

Table 3 presents the characteristics of bacterial enumeration in the operation unites of RPCM-WWTP. According to Table 3, raw wastewater had the most abundant bacterial community with a mean value of 4 × 10^8^ CFU/mL.

**Table 3:** Bacterial community (CFU/mL) in RPCM-WWTP.

Sample	Bacterial community (CFU/mL)
Raw wastewater	4 × 10^8^
Internal circulation reactor (IC reactor)	3.6 × 10^8^
Oxidation ditch	6.5 × 10^7^
Return activated sludge	2.3 × 10^8^

RPCM, recycled paper and cardboard mill; WWTP, wastewater treatment plant.

The same bacterial morphotypes were observed in all samples using the Gram-staining method (rod-shaped). The isolated colonies were composed of gram-negative and positive heterotrophic bacteria. Table 4 provides the percentage distribution of cultured G (−) and G ( +) dominant strains in the operation units of RPCM-WWTP based on the Gram-staining method. The most percentage distribution of Gram-negative and Gram-positive bacteria was related to oxidation ditch and IC reactor unites, respectively (Table 4).

**Table 4:** The percentage distribution of cultured G (−) and G ( +) dominant strains in RPCM-WWTP.

Sample	The percentage distribution	
	G (−)	G ( +)
Raw wastewater	33.33	66.67
Internal circulation reactor (IC reactor)	25	75
Oxidation ditch	66.67	33.33
Return activated sludge	40	60

RPCM, recycled paper and cardboard mill; WWTP, wastewater treatment plant.

The biochemical characterization of the isolated Gram-negative dominant bacterial community is presented in Table 5. Based on the results, the biochemical characterization of isolated Gram-negative strains included negative tests of oxidase, gelatin, and indole, along with the positive test of citrate utilization. In addition, the motility test of strains AT-1, IC-2, and AT-3 was negative. Table 5 also provides the results of other biochemical tests, including triple sugar iron (TSI), lysine iron agar (LIA), phenylalanine deaminase (PAD), urea, H_2_S, and Methyl red/Voges Proskauer (MR/VP).

**Table 5:** Biochemical characterization of screened Gram-negative dominant bacterial community.

Characteristics	RW-2	AT-1	A.T-2	AT-3	AT-4	IC-2	AS-2
Shape	Rod	Rod	Rod	Rod	Rod	Rod	Rod
TSI	K/A^a^	A/A^b^, Gas	K/A	K/K ^c^	A/A, Gas	A/A, Gas	K/A
LIA	K/K	K/K	K/K	K/K	K/A	K/A	K/K
PAD	−^d^	−	−	+^e^	−	−	+
Urea	−	+	−	−	+	+	+
MR/VP	−	−/+	−	−	+	−/+	−
Gelatin	−	−	−	−	−	−	−
Oxidase	−	−	−	−	−	−	−
Citrate utilization	+	+	+	+	+	+	+
Motility	+	−	+	−	+	−	+
Indole	−	−	−	−	−	−	−
H_2_S	−	−	−	−	+	−	−

^a^Alkaline over acid; ^b^acid over acid; ^c^ alkaline over alkaline; ^d^negative; ^e^positive.

#### Molecular identification

**Figure 2:**
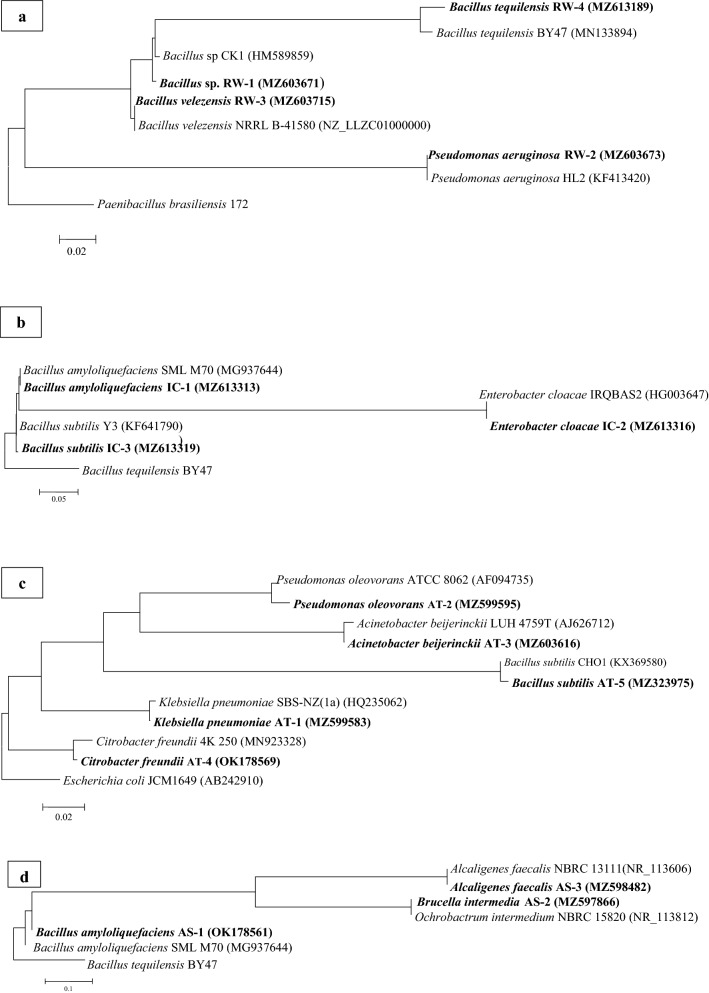
Phylogenetic tree of the dominant bacterial strains of RPCM-WWTP based on the partial 16s rRNA gene sequence representing the relationship between other neighboring members (the Genbank accession numbers were exhibited in parenthesis): (**a**) raw wastewater, (**b**) IC reactor, (**c**) oxidation ditch, and (**d**) return activated sludge. *Note*. RPCM: Recycled paper and cardboard mill; WWTP: Wastewater treatment plant; IC: Internal circulation.

In this study, a total of 15 dominant bacteria colonies were identified from raw wastewater, oxidation ditch, IC reactor, and return activated sludge, including 4, 3, 5, and 3 colonies, respectively. Figure 2 illustrates the BLAST analysis of the partial 16S rRNA sequence and phylogenetic tree of 15 isolated bacteria strains of the RPCM treatment plant. The BLAST analysis demonstrated 99–100% identity with the bacterial species of *Bacillus, Pseudomonas, Enterobacter*, *Alcaligenes, Citrobacter, Acinetobacter*, *Klebsiella*, and *Brucella*.

Based on the molecular characterization of isolated bacteria by 16S rRNA gene sequencing, most of the identified species belonged to the genus of *Bacillus* and *Pseudomonas*. In addition, the bacterial species of *Enterobacter*, *Alcaligenes, Citrobacter, Acinetobacter*, *Klebsiella*, and *Brucella* were identified (Fig. 2), which can be effective in the biodegradation of organic compounds such as COD, lignin, and color^24–26^. Moreover, they can be more effective in removing COD and color from RPCM wastewater by bioremediation. The use of foreign microorganisms for bioremediation has been successful, but their efficiency depends on different factors, including the ability to compete with indigenous microorganisms, predators, and various environmental factors^12,27^. Therefore, bioremediation by a dominant indigenous microbial community isolated from industrial wastewater that has been adapted to this environment can be a promising and effective method for the decomposition of wastewater pollutants. Additionally, previous studies highlighted the ability of some of these microorganisms to decompose various pollutants of municipal and industrial wastewater. For example, *Bacillus* species have shown promised applications for the degradation of industrial wastewater pollutants by bioremediation. Some other studies reported that certain microbial groups such as *Serratia liquefaciens, Bacillus megaterium*, and *Bacillus* sp. can be effective in removing resistant organic compounds from industrial wastewater^1^. In this study, indigenous bacterial strains including *K. pneumoniae* AT-1, *C. freundii* AT-4, and *B. subtilis* AT-5, were chosen for the bioremediation experiments of the RPCM effluent.

### Bioremediation experiments

#### Monitoring of RPCM effluent degradation by bacterial strains

The bioremediation experiments were conducted for 10 days on the RPCM effluent at pH = 7.2 in three setups, including effluent + culture, effluent + culture + mineral salt, and effluent + culture + mineral salt + glucose and the RPCM effluent treated with *K. pneumoniae* AT-1, *C. freundii* AT-4, and *B. subtilis* AT-5.

**Figure 3:**
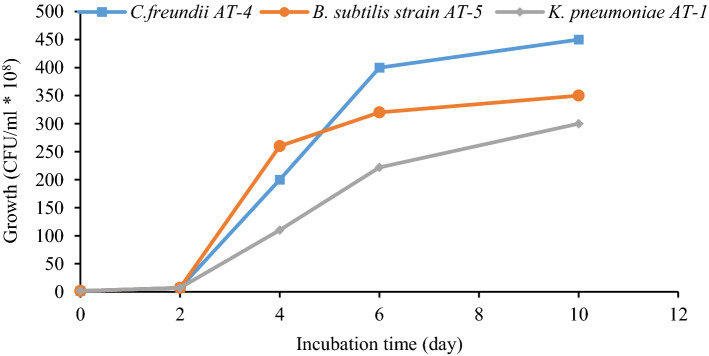
Bacterial culture growth during RPCM effluent bioremediation (effluent + culture + mineral salt + glucose). *Note*. RPCM: Recycled paper and cardboard mill.

In general, the bacterial growth during RPCM effluent treatment in setups revealed good growth at an incubation time of 2–4 days, and growth was significant up to six days of incubation. Figure 3 displays the bacterial growth in optimal conditions (glucose-containing setup). As shown, growth was considerable up to six days of incubation, and afterward, it demonstrated a low trend. Other researchers reported that the usage of glucose and sucrose (0.5–1.0%) as co-substrates is effective in the bacterial growth and treatment efficiency of the pulp and paper mill effluent^7,14^.

**Figure 4:**
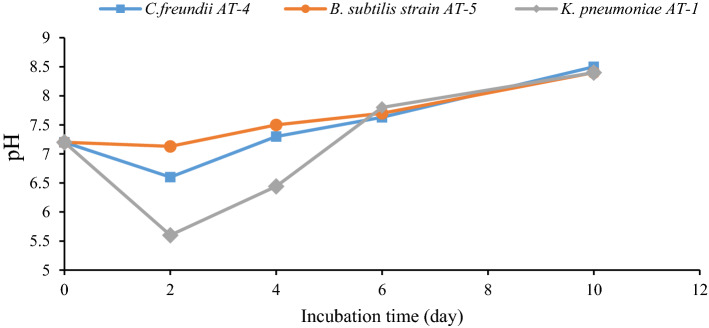
pH variations during RPCM effluent degradation (effluent + culture + mineral salt + glucose). *Note*. RPCM: Recycled paper and cardboard mill.

In optimal conditions with glucose, pH reduced up to 5.5, and then it slowly increased up to ~ 7.7 after six days of incubation and reached 8.5 at the end of the treatment (Fig. 4). In addition, pH variations in other setups had an increase of up to ~ 9 during four days of incubation and consequently reduced up to 8.7. The effluent pH changed during RPCM effluent degradation due to bacterial metabolic activities. In glucose-free cultures, a pH increase of up to ~ 9 occurred within four days of incubation. The effluent was treated at neutral pH; some bacteria can increase the pH of the environment due to the consumption of the available organic matter in the effluent as a carbon source, leading to the generation of alkali enzymes^28^. However, a decrease in the pH value was observed in the glucose-containing setup. The acidic pH might be due to glucose consumption in the effluent, resulting in the generation of acidic products^29^. Moreover, the partial fermentation process led to the generation of the acidic compound^11,29^. As the simple form of carbon sources (i.e., glucose) decreased, the substrate shifted from glucose to the organic carbon source and metabolic products, resulting in a gradual increase in pH^8^.

In the first two days, COD and color decreased due to glucose consumption, but subsequently, heterotrophic bacteria were forced to consume organic compounds, leading to a significant reduction in COD and color. This finding is in accordance with the obtained results from the glucose-free setup.

**Figure 5:**
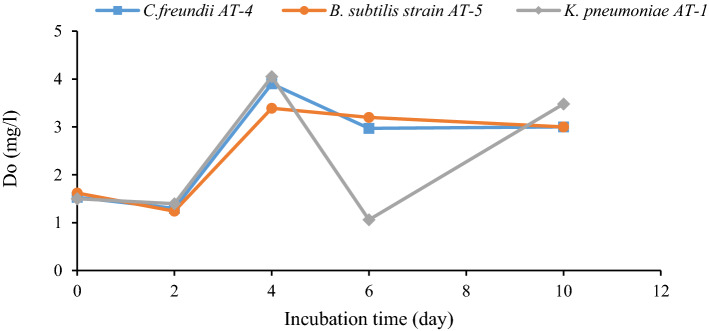
DO variations during RPCM effluent degradation (effluent + culture + mineral salt + glucose).

DO was one of the other measured factors during bioremediation (Fig. 5). The minimum DO was observed in the *K. pneumoniae* AT-1 culture after six days of incubation (1 mg/L). During RPCM effluent treatment, DO was reduced at the initial phase of bacterial growth due to the consumption of DO present in the effluent. The minimum dose of DO was 1 mg/L in the culture of *K. pneumoniae* AT-1 at six days of incubation.

#### Effect of additional co-substrate on COD and color biodegradation in the RPCM effluent

To confirm whether the effluent alone can be a contributor to bacterial growth and removal of COD and color, the experiments were conducted in the effluent and bacterial culture of *K. pneumoniae* AT-1, *C. freundii* AT-4, and *B. subtilis* AT-5. The bacterial growth was slow in the effluent alone, but bacteria could utilize the organic compounds of the RPCM effluent for growth.

**Figure 6:**
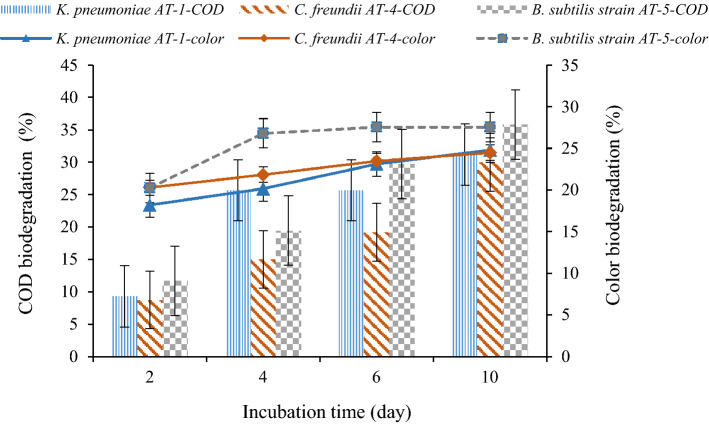
COD and color biodegradation rate by *K. pneumoniae* AT-1, *C. freundii* AT-4, and *B. subtilis* AT-5 during the incubation period (effluent + culture).

COD and color biodegradation rates, as the main factors of RPCM wastewater industries, are displayed in Fig. 6. Based on the results, the biodegradation of COD and color by the *B. subtilis* strain AT-5 after a 10-day incubation period was 35.8 ± 6.16% and 27.53 ± 3.96%, respectively. The biodegradation of COD and color by *K. pneumoniae* AT-1 and *C. freundii* AT-4 were 31.21 ± 4.3% and 24.8 ± 2.87%, as well as 30 ± 5.39% and 24.47 ± 3.1%, respectively. According to the results of the first setup without adding the co-substrate, the removal of COD and color by the *B. subtilis* strain AT-5 was higher compared to the other strains. These results indicate that *K. pneumoniae* AT-1 and *C. freundii* AT-4 strains can also degrade some of COD and color without using the co-substrate. However, previous research reported that the bacterial culture cannot grow and remove pollutants without adding carbon and nitrogen sources^8^.

**Figure 7:**
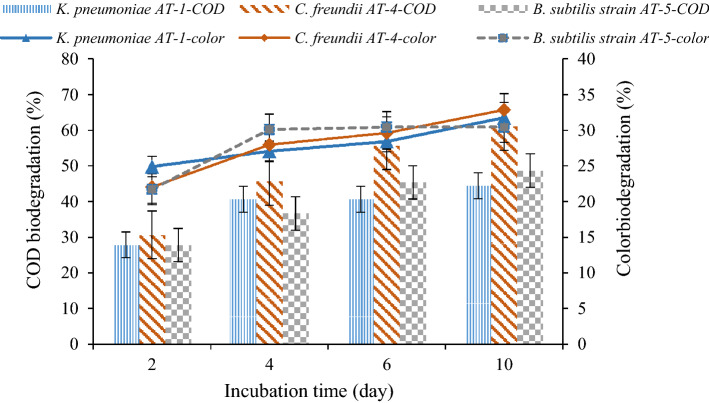
COD and color biodegradation rate by *K. pneumoniae* AT-1, *C. freundii* AT-4, and *B. subtilis* AT-5 in different incubation periods (effluent + culture + mineral salt).

Figure 7 depicts the effect of mineral salt on the performance of bacterial strains. As shown, the biodegradation of COD and color by *C. freundii* AT-4 after the incubation period of 10 days reached 61.10 ± 11.3% and 32.84 ± 4.4%, respectively. COD and color biodegradation by *K. pneumoniae* AT-1 and *B. subtilis* strain AT-5 were 44.47 ± 5.5%, 31.75 ± 3.66%, 48.68 ± 10.9%, and 30.43 ± 3.12%, respectively. In the second setup, the biodegradation of COD and color increased by all three bacteria (Fig. 7). In the case of *C. freundii* AT-4, the addition of the mineral salt had a significant effect on the growth and COD removal compared to the setup without the mineral salt. *C. freundii* AT-4 demonstrated the maximum COD biodegradation of 61.1%, while the maximum COD biodegradation of *C. freundii* AT-4 in culture without the mineral salt was 30.5%. *C. freundii* AT-4 had more COD and color biodegradation compared to the other two bacteria.

**Figure 8:**
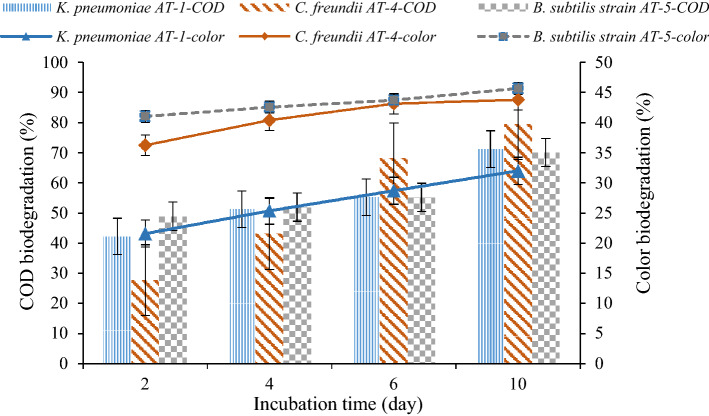
COD and color biodegradation rate by *K. pneumoniae* AT-1, *C. freundii* AT-4, and *B. subtilis* AT-5 in different incubation periods (effluent + culture + mineral salt + glucose).

Figure 8 illustrates the results of the simultaneous effect of the mineral salt and glucose as the co-substrate compound. According to the findings, by adding mineral salt and glucose, the biodegradation of COD and color by *C. freundii* AT-4 after the incubation time of 10 days reached 79.54 ± 7.88% and 43.81 ± 6.46%, respectively. COD and color biodegradation by *K. pneumoniae* AT-1 and *B. subtilis* strain AT-5 were 71.26 ± 6.97%, 32.01 ± 3.21%, 70.08 ± 8.37%, and 45.98 ± 4.31%, respectively.

Based on these results, the presence of glucose as an additional carbon source had a significant effect on bacterial growth and COD removal by all three bacteria. *C. freundii* AT-4 had more COD biodegradation in comparison to *K. pneumoniae* AT-1 and *B. subtilis* strain AT-5. In the case of color biodegradation, *B. subtilis* strain AT-5 and *C. freundii* AT-4 were more effective in removing color compared to *K. pneumoniae* AT-1. The results of this study indicated that in the presence of the mineral salt and glucose, the indigenous bacteria of RPCM-WWTP have more ability to degrade the COD and color of the effluent. Similar findings were reported in the other research studies^7^.

These results represented that the bacteria used glucose and glucose along with color, respectively, leading to a decrease in the color concentration of the effluent. One study reported similar results during the bacterial treatment of the effluent^29^. In another study, adsorption on the cell surface has been explained as a contributor to color removal^9^.

According to the obtained data, the indigenous bacteria of RPCM-WWTP were capable of significantly removing COD and color. Tiku et al. found that the usage of glucose and sucrose (0.5–1.0%) as co-substrates was effective in the bacterial growth and removal efficiency of the pulp mill effluent. However, additional sources of carbon have not been employed by the researchers due to excessive growth of biomass, leading to high turbidity in the samples^7^. Likewise, Tyagi et al. applied a bacterial-fungal consortium (*Micrococcus luteus*, *Bacillus subtilis*, and *Phanerochaete chrysosporium*) to remove biochemical oxygen demand (BOD), COD, and lignin from pulp and paper wastewater and reported biodegradation rates of 87.2%, 94.7%, and 97%, respectively^16^. The efficiency of *Aneurinibacillus aneurinilyticus, Paenibacillus* sp*.*, and *Bacillus* sp. was investigated in treating the paper mill effluent. Based on the results, COD removal by these bacterial strains was obtained 52–78% after six days of incubation^25^. In the treatment of the pulp paper mill effluent, *Bacillus cereus* and *Serratia marcescens* demonstrated biodegradation of color (45–52%), lignin (30–42%), BOD (40–70%), COD (50–60%), and phenol (32–40%), in a 7-day incubation period^26^.

#### Effect of the incubation period on the COD and color biodegradation of the RPCM effluent

Given that the retention time is an important factor for the effective wastewater treatment of RPCM, this study investigated the effect of the incubation period (2, 4, 6, and 10 days) on the removal of COD and the color of the effluent in three setups (Figs. 6, 7 and 8). In general, during effluent treatment, increasing the incubation period increased the biodegradation of COD and color. An increase in the incubation period led to an increase in the COD removal rate up to 79.55%, corresponding to *C. freundii* AT-4. This fluctuation was 70% and 55.3% for *Bacillus subtilis* strain AT-5 and *K. pneumoniae* AT-1, respectively. However, during 6–10 days of incubation, there were insignificant rates in setups 1 and 2 and an increase of about 15% in the third setup.

In color biodegradation, the experiment results were similar to the previous stage. Increasing the incubation period to six days caused a significant increase in biodegradation efficiency, but there was no significant increase after six days of incubation. The maximum biodegradation of color after six days of incubation in optimal conditions was obtained by *C. freundii* AT-4 (43.8%), *Bacillus subtilis* strain AT-5 (45.7%), and *K. pneumoniae* AT-1 (32.02%), respectively. Previous studies have reported the incubation periods of 6^8,14^, 7^26,30^, 9^11^, and 10^31^days for the removal of color from the pulp and paper effluent by different bacterial and fungal strains. In other studies, the removal rate of COD was 94.7%, 85%, and 78% by a bacterial-fungal consortium after an incubation period of nine days^16^, *Serratia liquefaciens* strain LD-5 with an incubation period of six days^8^, and *Paenibacillus* sp. strain LD-1 isolated from the soil sample after an incubation period of six days, respectively^14^. Table 6 summarizes the comparison results between the maximum biodegradation of COD and color by indigenous bacterial strains used in this study and other bacterial strains reported in similar studies.

**Table 6:** The maximum COD and color biodegradation by different bacterial strains.

Pollution supply	Pollutant	Bacteria	Incubationperiod (day)	Biodegradation rate (%)	References
Pulp and paper mill	COD, color	*Paenibacillus* sp, *Aneurinibacillus aneurinilyticus*, *Bacillus* sp.	6	52–78, 39–61	^25^
Pulp and paper mill	COD, color	*Paenibacillus* sp*.*	6	68, 78	^14^
Model and Kraft lignin from pulp paper	Color (model)	*Citrobacter freundii*	6	64, 50	^29^
	Color (Kraft lignin)	*Serratia marcescens*	6	60, 55	
Recycled paper and cardboard mill	COD	*C. freundii* AT-4	6, 10	68.2, 79.54	This study
	Color		6, 10	43.15, 43.81	
Recycled paper and cardboard mill	COD	*Bacillus subtilis* AT-5	6, 10	55.21, 70.08	This study
	Color		6, 10	43.73, 45.69	
Recycled paper and cardboard mill	COD	*K. pneumoniae* AT-1	6, 10	55.3, 71.26	This study
	Color		6, 10	28.71, 32.06	

Table 6 provides the maximum biodegradation of COD and color by *C. freundii* AT-4, *Bacillus subtilis* strain AT-5, and *K. pneumoniae* AT-1 used in the present study compared to other bacteria reported in the literature. Based on the findings, the maximum biodegradation of COD and color by indigenous bacterial strains was promising. Therefore, these bacterial strains can be applied for the removal of COD and color from RPCM wastewater. Given that these strains are part of the microbial consortium of RPCM wastewater and are adapted to the environmental conditions of WWTP, they can be suitable alternatives for the efficiency improvement of WWTP under optimal conditions.

## Conclusion

Overall, the promising biodegradation of RPCM wastewater treatment was investigated via the isolation and cultivation of its indigenous bacteria after the identification of *K. pneumoniae* AT-1, *C. freundii* AT-4, and *B. subtilis* strain AT-5 used for bioremediation experiments. Native strains could degrade contamination based on the COD and color of RPCM industry wastewater. Under optimal conditions, the maximum biodegradation of COD and color by *C. freundii* AT-4 was 79.54% and 43.81% after 10 days of incubation, respectively. Regarding *B. subtilis* strain AT-5 and *K. pneumoniae* AT-1, the maximum COD and color biodegradation was obtained at 70.08% and 45.96%, as well as 71.26% and 32.06%, respectively. The isolated strains of *K. pneumoniae* AT-1, *C. freundii* AT-4, and *B. subtilis* strain AT-5 were capable of COD and color degradation from the RPCM effluent under optimal conditions. The obtained results under optimal conditions were highly considerable in comparison with the efficiency results obtained from the oxidation ditch treatment unit in full-scale RPCM-WWTP. Therefore, these strains can be as promoting candidates for the bioremediation of RPCM-WWTP.

The results of this study can be helpful for microbial management strategies and increase the efficiency and stability of the treatment process. To obtain more detailed results, it is required to conduct further studies using other variables affecting the inoculation process to treat this type of wastewater.

## Data Availability

The datasets generated and analyzed during the current study are available in the (http://www.ncbi.nlm.nih.gov) repository [accession numbers MZ613189, MZ603671, MZ603715, MZ603673, MZ613313, MZ613316, MZ613319, MZ599595, MZ603616, MZ323975, MZ599583, OK178569, MZ598482, MZ597866 & OK178561].

## Abbreviations

AS: Activated sludge
AT: Aeration tank
BOD: Biochemical oxygen demand
COD: Chemical oxygen demand
DO: Dissolved oxygen
EC: Electrical conductivity
IC reactor: Internal circulation reactor
IC: Internal circulation reactor
LIA: Lysine iron agar
MR/VP: Methyl red/Voges Proskauer
OLR: Organic loading rate
PAD: Phenylalanine deaminase
RPCM: Recycled paper and cardboard mill
RPCM-WWTP: Recycled paper and cardboard mill wastewater treatment plant
RW: Raw wastewater
TSI: Triple sugar iron
TSS: Total suspended solids
